# An In-Vitro Analysis of Microleakage of Self-Adhesive Fissure Sealant vs. Conventional and GIC Fissure Sealants

**DOI:** 10.3390/dj7020032

**Published:** 2019-03-28

**Authors:** Kristina Gorseta, Ali Borzabadi-Farahani, Tara Vrazic, Domagoj Glavina

**Affiliations:** 1 Department of Pediatric and Preventive Dentistry, School of Dental Medicine, University of Zagreb, Gunduliceva 5, 10 000 Zagreb, Croatia; kgorseta@sfzg.hr (K.G.); glavina@sfzg.hr (D.G.); 2 Department of Clinical Sciences and Translational Medicine, University of Rome Tor Vergata, 00183 Rome, Italy; 3 Finchley Orthodontics, North Finchley, London N12 9EN, UK; 4 Private Practice, 42000 Varazdin, Croatia; vrazictara@gmail.com

**Keywords:** fissure sealants, marginal leakage, self-adhesive composite, GIC sealant, thermo-light curing

## Abstract

Background: The microleakage of a self-adhesive composite, a glass ionomer fissure sealant and a conventional resin-based fissure sealant were compared. Materials and methods: Fifty intact human molars with well-delineated pits and fissures were used and divided into 5 groups (*n* = 10). Group 1 specimens were etched (37% phosphoric acid) and sealed with conventional resin-based sealant (Helioseal F, Ivoclar Vivadent). Both Group 2 and 3 specimens were sealed with self-adhesive composite (Constic, DMG), but in Group 3, specimens were also etched (37% phosphoric acid). In Groups 4 and 5, specimens were sealed with a GIC sealant (Equia Fill, GC Company), but Group 5 was also exposed to thermo-light curing (TLC) with a LED polymerization unit (60 s). Subsequently, specimens were thermocycled (1800 cycles, dwelling time of 10 s), immersed in 50% silver nitrate solution (45 min), placed in a photo-developing solution (Kodak) under a lamp (120 W, 6 h), and cut into 3–4 slices. Marginal leakage (dye penetration depth) was evaluated under a light microscope and the worst score of each specimen was recorded (0–3). Results: Constic showed the lowest microleakage (Constic: 80% scored 0 or 1), followed by Helioseal (30% scored 0 or 1) (*p* = 0.037). Microleakage in groups sealed with Constic (with and without etching) were not different (*p* = 0.473). The quality of seal deteriorated after etching when Constic was used. However, TLC improved the seal when GIC sealant was used (*p* = 0.016) and also in comparison to Helioseal (*p* = 0.004). The TLC GIC sealant (Equia Fill, 90% scored 0 or 1) performed well, similar to self-adhesive composite (Constic, 80% scored 0 or 1) (*p* = 0.206). Conclusion: The present findings suggest that the self-adhesive sealant and the GIC sealant that were exposed to TLC had comparable sealing ability and superior sealing characteristics compared to the conventional resin-based sealant. A long-term clinical trial is needed to assess the intra-oral performance.

## 1. Introduction

Recent systematic reviews suggest that fissure sealing has been effective in caries prevention and we know that at 24 months, resin-based sealants reduced caries by 11–51%, compared to no sealant usage. However, there is a scarcity of evidence on relative effectiveness of resin-based sealants, glass ionomer sealant and other types of sealants when compared to each other [1,2]. Microleakage of dental restorative materials is the main problem in clinical dentistry and is defined as leakage of bacteria, liquids, molecules and ions between tooth and restorative materials.

Adequate adhesion at the interface between hard dental tissues and restorative materials is crucial for achieving good clinical performance and durable restoration [3]. Leakage could lead to dental sensitivity, tooth discoloration, and development of secondary caries.

The major disadvantage of resin-based sealants is the technical sensitivity during clinical applications. Continuous improvement of materials and clinical techniques has led to better marginal adaptation. Marginal leakage is a side effect of unsuccessful treatment and is often used as a measure to predict clinical performance of the material. The adhesion in self-adhesive composites (SAC) is based on the self-etching approach, and the three traditional steps of adhesion (etching, priming, and bonding), are accomplished by a single application of a solution [3]. Reducing clinical steps in application of adhesive eliminates the possibility of cavity contamination and over-drying/wetting issues. Further, flowable composites have been reported to improve marginal adaptation of restorations in relation to their rheological properties [4,5,6]. Combining an all-in-one bonding system and a flowable composite holds great potential with respect to saving chair time and minimizing handling issues.

There are a few investigations on the adhesive properties of these simplified self-adhesive fissure sealant composite materials [7,8,9]. Some studies reported that SAC achieved lower bond strength compared to the traditional flowable composites that were used with bonding agents [8]. According to the manufacturer, Constic, a self-etching and self-adhesive flowable composite, combines an etching gel, bonding agent and flowable composite.

The 10-methacryloyloxydecyl dihydrogen phosphate (MDP) monomer in Constic, was found to form sTable 10-MDP-Ca salts, leading to a strong chemical bond with hydroxyapatite [10]. The literature suggests that many confounders and variables influence the microleakage in dental materials [11]; however, it is still widely used as an indicator of the materials’ sealing ability [1,2,3,11].

The effect of thermo-light-curing (TLC) on the physical properties of glass ionomer cements has been investigated previously [12,13]. This study investigated the marginal adaptation and retention rate of a self-adhesive composite with different enamel pre-treatment compared to a glass ionomer sealant material with a different setting procedure (chemical setting and setting ‘on command’ with thermo-light-curing with LED polymerization unit).

The objective of the present study was to evaluate the effect of different enamel treatments on the microleakage of self-adhesive flowable composites (Constic, DMG, Hamburg, Germany) in comparison with a conventional resin-based sealant with classic enamel etching prior to treatment.

The null hypotheses that were tested were as follows:

1. Marginal adaptation of a self-adhesive composite is similar to the conventional resin-based fissure sealant.
2. Pre-treatment of enamel (etching) has no influence on microleakage of a self-adhesive composite.
3. Marginal adaptation of a self-adhesive composite is similar to the thermo-light cured glass ionomer fissure sealant.

## 2. Materials and Methods

### 2.1. Sample Preparation

A total of 50 intact, non-carious human molars, extracted for orthodontic reasons, were included in this study. Prior to the experiment, teeth were cleaned under running water to remove organic residues, decontaminated with 0.5% Chloramine-T compound, and stored in distilled water.

Specimens were randomly divided into five equal groups (*n* = 10), according to the tested materials. The control group (G1) was sealed with conventional resin-based sealant (Helioseal F, Ivoclar Vivadent AG, Schaan, Liechtenstein) with prior enamel etching (37% phosphoric acid). The Group 2 (G2) specimens were sealed with self-adhesive composite resin (Constic, DMG, Hamburg, Germany) without enamel pre-treatment according to the manufacturer’s instructions. In Group 3 (G3), the enamel was etched with 37% phosphoric acid and sealed with the Constic. The Group 4 specimens (G4) were sealed with glass ionomer (Equia Fill, GC Company, Japan) and covered with Equia varnish. Finally, Group 5 (G5) was sealed with glass ionomer (Equia Fill, GC Company, Tokyo, Japan) and thermo-light-cured with LED polymerization unit (Bluephase G2, Ivoclar Vivadent AG, Schaan, Liechtenstein) for 60 s.

### 2.2. Specimen Aging

To simulate the aging of material, each of the 5 groups were aged according to the following protocol: after 1-day of storage in distilled water, the specimens were subjected to thermocycling for 1800 cycles between 5 °C and 55 °C with a dwell time of 10 s in each bath and a transfer time of 5 s.

### 2.3. Assessment of Microleakage

After thermocycling, the root apices of the teeth were sectioned off 2 mm beneath the cemento-enamel junction with a diamond disk (Diamond Cut-off Wheel M1D13, Struers, Ballerup, Denmark). The tooth crowns were then sealed with a composite resin (Tetric EvoCeram, Ivoclar Vivadent) to prevent infiltration of the dye solution through this area. The entire surface of each tooth was covered with two layers of nail varnish, leaving a 1 mm uncovered area around the margins of the fissure sealant. This cover is crucial to avoid dye penetration to other parts of the tooth. Specimens were prepared for microleakage testing by coating the surface with two layers of fast-setting nail varnish within 1 mm of the bonded interface.

The sealed specimens were then immersed in a 50% weight silver nitrate dye solution for 24 h at room temperature [14]. The silver-impregnated teeth were then rinsed with distilled water and immersed in photo-developing solution for eight hours (Dental X-Ray Developer, Kodak Co, Rochester, NY, USA).

**Figure 1:**
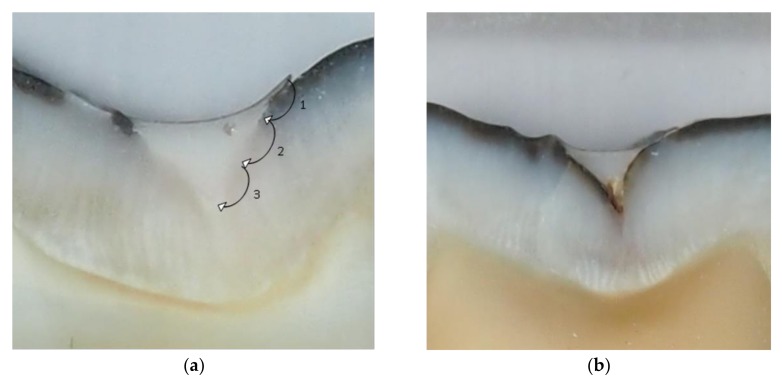

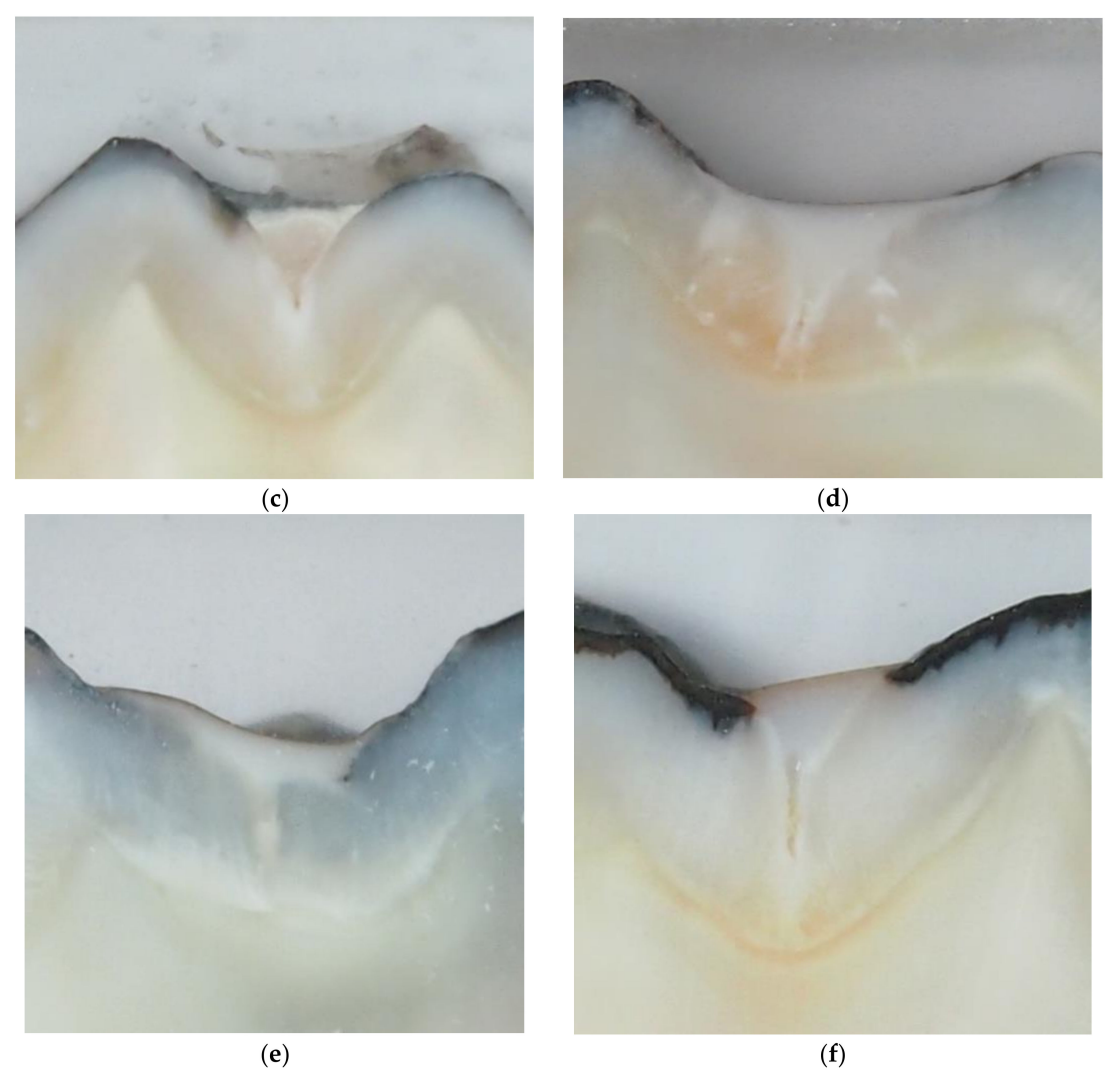
Score scheme of dye penetration at the material/tooth interface (**a**): 0-no evidence of dye penetration; 1-dye penetration of less than 1/3 from the margin of restoration; 2-dye penetration of more than 1/3 and less than 2/3 from the margin of restoration; 3-dye penetration of more than 2/3 from the margin of restoration. Examples of specimens sealed with Helioseal F (**b**); Equia + varnish (**c**); Equia thermo-light-cured for 60 s (**d**); Constic following phosphoric acid etching (**e**); and Constic (**f**).

After rinsing with water, the teeth were placed in acrylic resin (Citofix Kit, Struers A⁄S, Ballerup, Denmark) and then sectioned buccolingually using a diamond cutting saw (Minitom, Struers A⁄S, Ballerup, Denmark) operating at a speed of 125 rpm with an applied load of 100 g in 2–3 sections. Sectioned specimens were evaluated under a stereomicroscope (Opton STEMI SV8, Oberkochen, Germany) at 2.5× magnification and photographed (Artray ARTCAM 300MI). Marginal leakage was assessed using an Olympus DP soft, Version 3.2. Evaluation of the marginal adaptation was performed based on the Oberholzer criteria [15] (Figure 1).

### 2.4. Assessment of Sealant Retention and Microleakage (Dye Penetration)

After thermocycling, the sealant retention was evaluated. A calibrated examiner was passed with a 0.5 mm diameter probe along the margins of the sealant to verify continuity, integrity, failure or loss of sealant. Classification of sealant retention was based on Simonsen’s criteria [16] as follows:

1. Complete retention.
2. Partial loss.
3. Complete loss.

Microleakage was assessed on a digital image of each section, acquired using a digital photo camera (Olympus). The sectioned restorations were also examined under a stereomicroscope (Olympus) at 2.5× magnification. Dye penetration at the material/tooth interface of the enamel was scored (Figure 1) as follows (Oberholzer et al.) [15]:

0-no evidence of dye penetration;
1-dye penetration of less than 1/3 from the margin of restoration;
2-dye penetration of more than 1/3 and less than 2/3 from the margin of restoration;
3-dye penetration of more than 2/3 from the margin of restoration.

The worst scores of the microleakage per tooth were used for statistical analyses. Prior to evaluation, specimens were sectioned under a water spray parallel to the long axis (bucco-lingually) with a slow-speed diamond saw (Buehler, Inc., Jasper, IN, USA). Three to four slices were prepared from each tooth.

### 2.5. Statistical Analysis

The microleakage scores data were analysed using Statistica 7.0 Package.

Statistical analysis was performed using the Kruskal–Wallis non-parametric test and the Mann–Whitney U test (*p* < 0.05) for post-hoc analysis. The chi-square test was also used to compare the dye penetration score in different groups.

## 3. Results

Results showed 100% retention rate of all tested fissure sealant after the thermocycling procedure. Distribution of the worst score per sealant is reported in Table 1. The best marginal adaptation was shown in the Equia Fil group used in combination with thermo-light curing. The difference between groups sealed with Constic with and without etching of enamel was not significantly different (Chi-square = 0.952; *df* = 1; *p* = 0.3291) (Table 2). Etching of the enamel did not affect the adhesion of self-adhesive composite resin (Table 2). Comparing the self-adhesive composite resin and conventional composite resins for fissure sealing, better marginal adaptation was obtained in the self-adhesive composite group (*p* < 0.05). Regarding the depth of dye penetration, significant differences were recorded between Helioseal F and Constic (Chi-square = 5.0505; *df* = 1; *p* = 0.0246). There was a statistically significant difference between thermo-light cured Equia Fill and Equia Fill applied with varnish (Chi-square = 7.50; *df* = 1; *p* = 0.0062). Equia Fill with thermo-light curing completely sealed the fissure system in most of the samples. Only the first null hypothesis was rejected (marginal adaptation of new self-adhesive composite is as good as conventional resin-based fissure sealant).

## 4. Discussion

Presently, there are two broad categories of fissure sealants based on light-cured, resin-based fissure sealants and glass ionomer sealants [17]. Sealant placement is very technically sensitive. Microleakage is still a major problem, and the primary reason for failure of composite resin restorations. There are a few studies on the microleakage of SAC and the effect of enamel etching on marginal adaptation [6,18,19,20]. The first marketed self-adhesive composite has shown improved benefits by treatment using phosphoric acid and laser irradiation [21,22]. The literature provides limited information on the microleakage characteristics of available self-adhesive composites and the effect of additional enamel pretreatment on sealing ability. The aim of this study was to evaluate the effect of enamel etching on the microleakage of SAC in comparison with a conventional resin based sealant with an etch-and-rinse system and in comparison with a glass ionomer fissure sealant.

Bektas et al. suggested that using an adhesive resin with self-etching flowable composite could increase dentin bond strength and reduce microleakage to hard dental tissue [6]. However, in other studies, it has been found that there was no significant difference detected when a self-adhering flowable composite was applied, according to the manufacturer’s instructions, without pretreatment [18,23]. In the present study, differences between groups sealed with Constic with and without etching of enamel were not significantly different.

Constic is a self-adhering, flowable fissure sealant composite resin, with a bonding system that relies on the adhesive monomer glycerol phosphate dimethacrylate (GPDM). Acid etching is possible due to the phosphate group of GPDM.

Etching of the enamel, proposed in etch and rinse systems, increases the surface energy of the enamel surface by removing the smear layer. This step is not necessary when SAC is applied and it does not have an influence on marginal adaptation [8]. This fact is very important in everyday clinical practice in paediatric dentistry.

The glass ionomer with heating showed slight nano-leakage compared with all other tested groups. This could be related to the fact that the glass ionomer chemically bonds to hard dental tissue. Gorseta et al. showed that heat application during the setting of GIC improved the adhesion to enamel [24]. Adding external energy during the setting of GIC increases kinetic energy inside the material. This leads to better connection with hard dental tissue and lower penetration of silver ions [24]. This is in accordance with findings obtained in this in-vitro study.

The greatest leakage was observed in the Helioseal F group. Inadequate isolation increases the risk of microleakage and subsequent treatment failure. Therefore, self-adhering materials have become popular due to easier application and fewer working steps. This is very important in paediatric dentistry as poor cooperation of children and more steps in the application lead to greater possibility of failure.

It should be noted that presently there is no standardized method for the in vitro evaluation of microleakage of fissure sealants, making it challenging to compare present findings with previous studies [17,25]. We expressed microleakage as the score of penetration of dye along the enamel-sealant interface of fissures, and used a validated scoring system [15,17]. However, it has been suggested that evaluating the percentage of penetration of the dye along the enamel-sealant interface of pits and fissures would be more accurate than the use of dichotomous (presence or absence) or numerical scales, [17] which is a matter for future studies. We should add that presently no fissure sealant retains the sealing ability over time, and all eventually show some degree of microleakage [25]. This is partly due to differences between the coefficient of thermal expansion of sealants and that of enamel [25]. Therefore, long-term clinical trials are needed to recommend the best sealant. Under the limitations of this study, it can be concluded that there was no beneficial effect of enamel pre-treatment (etching) on the sealing ability of self-adhering composites.

## Figures and Tables

**Table 1:** Frequency table of the worst score per sealant (0-no evidence of dye penetration; 1-dye penetration to less than one third from the margin; 2-dye penetration up to two thirds from the margin; 3-dye penetration up to bottom of fissure).

Maximum Dye Penetration Score	G1, Helioseal F	G2, Constic	G3, Constic + Etch	G4, Equia + Varnish	G5, Equia + Thermo-Light Curing
0	1	4	3	2	7
1	2	4	1	1	2
2	1	0	1	3	0
3	6	2	5	4	1
Total	10	10	10	10	10

**Table 2:** Mann–Whitney U Test Post-hoc analysis of multiple comparisons of different sealants.

	Helioseal F	Constic	Constic + Etching	Equia Fill + Varnish	Equia Fill + Heat (TLC) *
Helioseal F	-	0.037	0.505	0.489	0.004
Constic	0.037	-	0.473	0.116	0.206
Constic + etching	0.505	0.473	-	0.388	0.071
Equia Fill + varnish	0.489	0.116	0.387	-	0.016
Equia Fill + heat	0.004	0.206	0.071	0.016	-

* Thermo-Light-Curing.
